# Integrating machine learning and multi-criteria decision analysis for health risk management in water distribution networks

**DOI:** 10.1038/s41598-026-52465-z

**Published:** 2026-05-15

**Authors:** Uchit Sangroula, Victor Viñas, Michael Odhiambo, Thomas J. R. Pettersson

**Affiliations:** 1 Department of Architecture and Civil Engineering, Water Environment Technology, Chalmers University of Technology, Gothenburg, SE-412 96 Sweden; 2 AFRY AB, Grafiska vägen 2A, Gothenburg, SE-412 83 Sweden; 3 Department of Urban Water Engineering, Norconsult AB, Gothenburg, SE-402 76 Sweden

**Keywords:** Water distribution networks, Water safety plan, Health risks, Decision making, Civil engineering, Environmental sciences, Risk factors

## Abstract

Leakages and breaks in water distribution networks (WDNs) cause significant water losses and pose health risks due to pathogen intrusion. The Water Safety Plan (WSP), developed by the World Health Organization (WHO), provides a comprehensive framework for identifying, assessing, and controlling risks within water supply systems. This study demonstrates the application of the WSP framework through a case study of a WDN in Sweden. Pipe break probabilities were estimated using three classification models: Logistic regression, random forest, and extreme gradient boosting (XGBoost), while hydraulic and health consequences were evaluated using hydraulic modelling and Quantitative Microbial Risk Assessment (QMRA) to quantify the overall health risk. A Multi-Criteria Decision Analysis (MCDA) approach, specifically the Technique for Order of Preference by Similarity to Ideal Solution (TOPSIS) was utilized to prioritize risk mitigation strategies through breakage and leakage control measures. The proposed approach integrates predictive modelling, consequence evaluation, and decision analysis, offering a structured method for water utilities in prioritizing interventions and improving the overall safety and reliability of WDNs.

**Supplementary Information:**

The online version contains supplementary material available at 10.1038/s41598-026-52465-z.

## Introduction

Water distribution networks (WDNs) are vital components of water supply systems necessary for providing consumers with sufficient water of appropriate quality^1^. Physical defects such as pipe breaks, cracks, and leakages are characteristic of aging water distribution infrastructure. Leakages and breaks in distribution pipelines result in significant water losses, causing substantial financial, environmental, and social impacts^2^. Globally, the volume of water lost due to leakages and breakages is estimated at 346 million cubic meters per day^3^. In Sweden, approximately 15–20% of the municipal drinking water is lost due to leakage and breakages^4^. Additionally, pipe breaks and leakages can serve as pathways for pathogen intrusion due to inadequate pressure, posing serious risks to human health^5^. Practical rehabilitation strategies are therefore necessary to maintain distribution pipelines and reduce associated losses and health risks^6^. Developing such strategies requires considering risks related to pipe breaks and leakage, budget constraints, and resource limitations.

The World Health Organization (WHO) has recognized the importance of ensuring water safety in distribution systems and subsequently developed the Water Safety Plan (WSP) framework^7^. The WSP encompasses a comprehensive risk assessment and management approach, where potential hazards within the water supply system are identified, and control measures implemented to prevent contamination. The principal objective of WSPs is to proactively protect public health by maintaining water quality from source to consumers. The preventive strategy of WSPs, which focuses on identifying and mitigating risks, has been recommended by WHO guidelines for the preventive management of WDNs^8^. WSPs have been successfully implemented worldwide, demonstrating significant benefits in reducing health risks^9^.

There is also an increasing need for integrated, data-driven, and stakeholder-inclusive approaches that enable water utilities to identify significant risks in their WDNs and select the most effective interventions. Previous studies have typically utilized multiple tools independently for prediction, risk assessment, and maintenance planning in WDNs. For example, some studies have employed machine learning (ML) for pipe break predictions^10^, others have applied Quantitative Microbial Risk Assessments (QMRA) for contamination evaluations^11^, and some have utilized decision-making methods for hazard prioritization^12^. However, an integrated approach that combines these elements remains limited in the literature.

This study proposes a framework that integrates predictive modelling, consequence evaluation, and decision analysis within the WSP framework. The study involves four distinct steps: (i) identifying vulnerable pipes and assessing their impact upon failure, (ii) conducting health risk assessments, (iii) identifying control measures, and (iv) using Multi-Criteria Decision Analysis (MCDA) to prioritize leakage and breakage control strategies for health risk management. The likelihood of pipe breaks (P) is estimated using multiple supervised ML classification models^13,14^. Consequences (C) are assessed by evaluating impacts on both human health and network performance, by combining QMRA^15^ and hydraulic modelling (EPANET 2.2)^16^. Risk assessment is performed by multiplying the probability (P) by the consequence (C). A GIS-based risk map is then developed to categorize pipes according to their risk levels within the network.

Subsequently, a series of interviews are conducted with water experts and stakeholders for the identification of different strategies for health risk reduction. A MCDA approach^17^ is utilized to determine the best strategies with regards to reducing health risks. The Technique for Order Preferences by Similarity to Ideal Solutions (TOPSIS) method^18^ is utilized for the MCDA. This approach is demonstrated through a case study on a local WDN in Skåne, Sweden. The novelty of this work lies in uniting predictive modelling, microbial risk analysis, and multi-criteria decision support into one integrated framework. The results from this study can help water utilities prioritize pipe renewal and rehabilitation and proactively address vulnerabilities and health risks in WDNs.

### Background

#### Risk assessment

A risk assessment is a structured process that identifies both the probability of occurrence of an undesired event and the extent of the adverse consequences that may arise from that event^19^. For a WDN risk assessment, the general term for risk is expressed as Eq. (1).

1 $$\:R = P \cdot C$$

where R is the risk, P is the likelihood of occurrence of an undesired event (pipe break) and C represents the consequences of the event (associated impacts).

##### Probability of pipe breaks

Numerous studies have investigated pipe break probabilities in WDNs^20,21^, categorizing pipe break causes into physical, environmental, and operational factors^22^. Common physical factors include pipe length, diameter, material, and age. Environmental factors often include traffic loads, soil types, and climate conditions, whereas operational factors include internal pressure, past failures, and pump operations. Variables predicting pipe breaks are termed explanatory variables.

In general, three main prediction model types are utilized: physical^23^, statistical^24^, and ML models^25^. Physical models simulate mechanical and environmental stresses on pipes, but require extensive and costly pipe inspection data (field measurements)^26^. Statistical and ML models, being more cost-effective and data-efficient, predict failures based on historical data and mathematical relationships^27^. Recently, ML models have grown in popularity due to superior predictive accuracy and robust handling of complex data relationships^28,29^. Typical model outputs include failure probabilities, pipe classifications, time to failure, and failure rates.

##### Consequences of pipe breaks

Pipe breaks can result in a broad range of serious consequences such as water loss, high repair, and replacement costs, threats to human health, environmental damage and customer dissatisfaction^30^. Hydraulic and health consequences are particularly important as they directly affect system reliability and public safety. Hydraulic consequences involve network disruptions affecting water delivery, measured by disconnected nodes and unsupplied flow volume. Health consequences involve contamination risks, potentially leading to waterborne diseases^31–33^ and endemic illnesses globally^34,35^. Contaminants such as *Campylobacter*, *Salmonella*, and norovirus from pipe breaks can cause illnesses ranging from mild to severe^36^.

#### Multi-criteria decision analysis (MCDA)

Decision making is an integral part of any successful project. It can involve either individuals or groups and can be based upon a single criterion or take multiple criteria into account^37^. MCDA is a structured decision-making process which aims to prioritize alternatives while considering multiple varying criteria^38^. The typical MCDA process involves: (i) defining the decision problem, objectives, alternatives, and evaluation criteria; (ii) structuring the problem; (iii) incorporating stakeholder preferences; (iv) evaluating alternatives; and (v) analysing results.

MCDA methods can be categorized into Multi-Objective Decision Making (MODM), focusing on optimizing continuous variables, and Multi-Attribute Decision Making (MADM), which selects optimal discrete alternatives. Both methods have found extensive utility in the field of water supply management, including the design of distribution systems^39^, optimization of monitoring networks^40^, and maintenance planning to minimize risks^41,42^. Recent studies have highlighted the superior effectiveness of MADM methods over MODM approaches in water distribution system rehabilitation^43,44^. Common examples of MADM methods utilized for decision-making tasks include the Analytical Hierarchy Process (AHP)^45^, TOPSIS^18^, and Elimination and Choice Translating Reality (ELECTRE)^46^.

## Methods

**Fig. 1:**
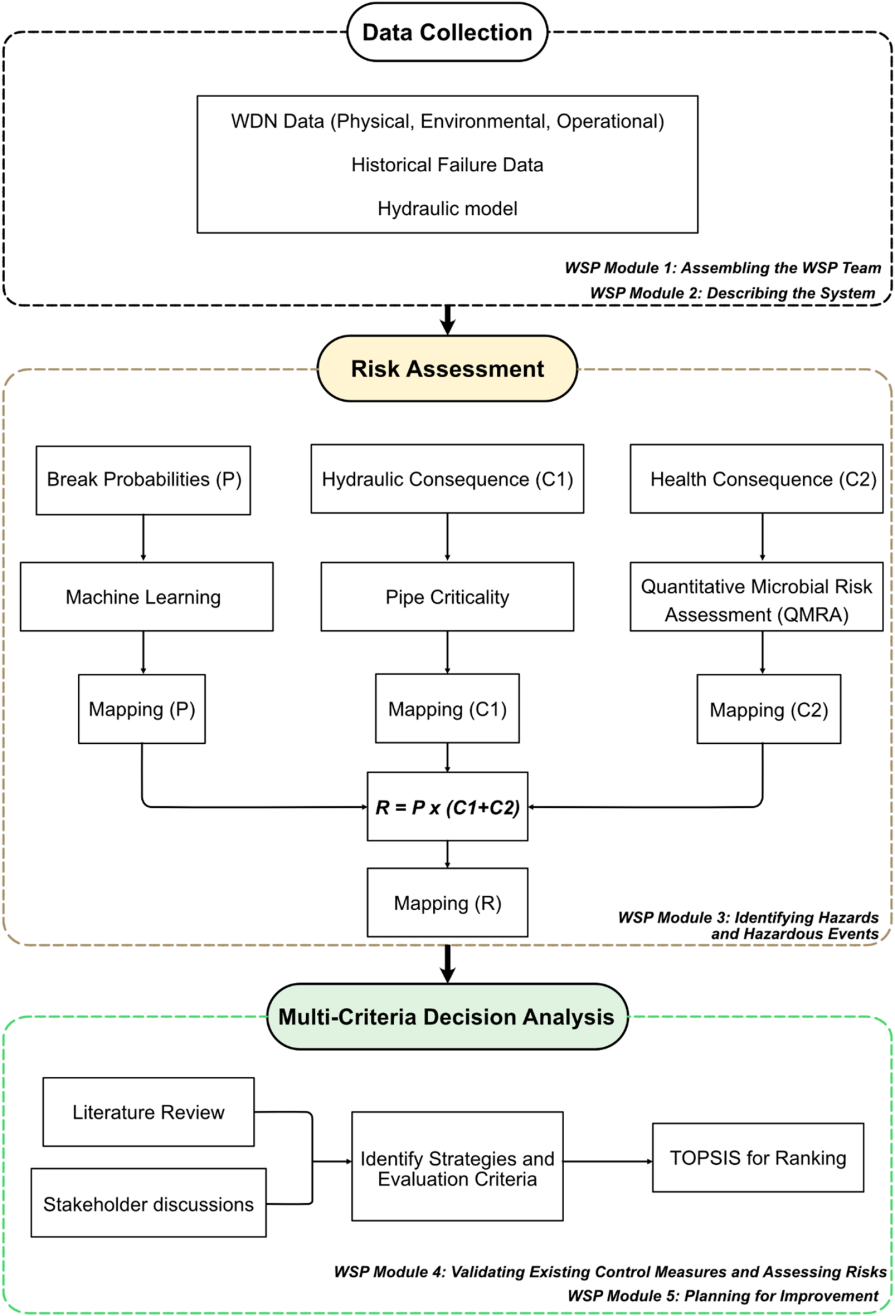
Overall structure of the study adapted from the WSP framework^7^.

The WSP framework consists of 11 key modules where each module builds upon the previous step. Figure 1 illustrates the specific WSP modules emphasized in this study.

### Case study description

This study focused on a small segment of a larger WDN located in Skåne County, southern Sweden, previously analysed by Odhiambo, et al. [11], hereafter referred to as the case study section.

This section spans 13.5 km, with pipe diameters ranging from 25 mm to 200 mm. The network is dominated by polyethylene (PE), which accounts for 41% of the total pipe length, followed by cast iron (36%) and ductile iron (11%), while the remaining materials together account for 12%. Based on the historical break record, the overall break rate in the case study section was 13.4 breaks/100 km/year. Among the dominant materials, ductile iron exhibited the highest break rate (24.3 breaks/100 km/year), followed by cast iron (18.5 breaks/100 km/year) and PE (10.2 breaks/100 km/year).

The annual water supply volume is approximately 50,000 m³, with losses due to pipe breaks and leakages estimated at 0.054 l/s/km, representing 27% of total water production. The pipes in this WDN are installed at depths of 1.5 to 2 m below the ground surface. Local staff indicated that most sewer pipes are positioned at the same elevation as drinking water pipes, with a horizontal separation of approximately 0.5 m.

### Risk assessment methodology

The risk assessment was done by considering both the likelihood of pipe breaks and their hydraulic and health consequences, as outlined in Eq. (2). Detailed descriptions of each step are provided in the sections below.

2 $$\:Risk = Likelihood\:of\:Break \cdot (Hydraulic\:consequence + Health\:consequence)$$

#### Break probabilities

Three ML models: logistic regression, random forest, and eXtreme Gradient Boosting (XGBoost) were used to estimate the probability of pipe break. Brief descriptions of these models are provided below.

##### Logistic regression

Logistic regression (LR) estimates the probability of a binary outcome by applying a logistic (sigmoid) function to a weighted sum of the input features. LR is mathematically formulated as Eq. (3) [47].

3 $$\:p = \frac{1}{{1 + e^{{ - (w_{o} + \sum \: _{{i = 1}}^{m} w_{i} \cdot x_{i} )}} }}$$

where *p* is the output probability of each sample, *x*_*i*_ denotes the value of the *i*^*th*^ feature, *w*_*i*_ is the weight of the *i*^*th*^ feature and *w*_*o*_ is the bias constant. The weighted sum of the features, combined with the bias, is passed through the sigmoid function, which transforms it into a value between 0 and 1; this value is then compared to a decision threshold to assign each pipe to the intact or break class.

##### Random forest

Random forest (RF) is an ensemble learning algorithm that estimates the probability of a binary outcome by constructing many decision trees and aggregating their predictions [48]. It is a bagging-based method, where each tree is trained on a different bootstrap sample of the training data, and at each split a random subset of features is considered. The predicted probability for an input feature vector *x* is obtained by averaging the probabilities from *B* trees, as shown in Eq. (4):

4 $$\:p=\frac{1}{B}\sum\:_{b=1}^{B}{p}_{b}\left(\mathrm{x}\right)$$

where *p* is the output probability for each pipe, *p*_*b*_*(x)* denotes the probability predicted by the *b*^*th*^ decision tree for the input feature vector *x*, and *B* is the total number of trees in the forest. The probabilities from all trees are averaged to produce a final value between 0 and 1; this value is then compared to a decision threshold to assign each pipe to the intact or break class.

##### XGBoost

Extreme gradient boosting (XGBoost) is an ensemble learning algorithm that estimates the probability of a binary outcome by building decision trees sequentially, where each new tree is trained to improve the predictions of the previous trees^49^. The model combines the outputs of *T* trees into a single score and then converts this score into a probability using the logistic (sigmoid) function, as shown in Eq. (5):

5 $$\:p=\frac{1}{1+{e}^{-f\left(x\right)}}\:,\:\:f\left(x\right)=\sum\:_{t=1}^{T}{g}_{t}\left(x\right)$$

where *p* is the output probability for each pipe, *x* is the input feature vector, *g*_*t*_*(x)* is the contribution of the *t*^*th*^ decision tree, and *f(x)* is the combined score from all trees. The sigmoid function transforms the score into a value between 0 and 1; this value is then compared to a decision threshold to assign each pipe to the intact or break class.

##### Data description and preparation

The explanatory variables (i.e., model features) are selected from three different categories i.e. physical, environmental, and operational. Table 1 provides an overview of the explanatory variables adopted for the model.

**Table 1:** Explanatory variables for the ML models.

Explanatory variable	Name	Type	Description
Physical	Length	Numerical	Pipe length in m
	Diameter	Numerical	Pipe diameter in mm
	Age	Numerical	Pipe age in years
	Material	Categorical	Material of pipe section
Environmental	Elevation	Numerical	Elevation from mean sea level
	Soil type	Categorical	Major soil type at pipe location
	Roads & Trainlines	Categorical	Proximity to the pipe
Operational	Pressure	Numerical	Average operational pressure
	Impact score	Numerical	Degree to which a pipe is affected by failures of other pipes in WDN
	Historical breaks	Numerical	Recorded breaks in the last 20 years

Physical pipe information and break records provided by the municipality were spatially matched using geoprocessing. The average operational pressure was simulated over a 24‑hour period. Soil data were obtained from the Geological Survey of Sweden (SGU), the national authority for geology and related geosciences. Proximity to infrastructure (roads and train lines) was determined by creating buffer zones around pipes and overlaying these with relevant spatial datasets.

In this study, an ‘Impact score’ feature was developed to capture how significantly each individual pipe is affected by the failures occurring elsewhere in the distribution network. To achieve this, a comprehensive hydraulic modelling analysis was conducted, in which failure was simulated in each pipe of the network systematically. For each simulated pipe failure, the resulting changes in pressure and flow across the entire distribution network were monitored. This analysis enabled the quantification of how sensitive each pipe is to hydraulic disturbances caused by failures occurring elsewhere in the network. The cumulative hydraulic impact experienced by each pipe across all simulations was normalized and squared to derive the ‘Impact score.’ Pipes receiving higher impact scores indicate those pipes which are more frequently and severely affected by failures occurring elsewhere in the network.

Previous studies that have addressed spatial or network-level dependencies in a WDN by employing clustering methods or hotspot analyses, such as spatial clustering or k-means segmentation^50,51^, have relied on historical failure patterns or physical characteristics. In contrast, the impact score dynamically captures the spatial relationships and interdependencies among pipes within the WDN.

The ‘Historical breaks’ variable was used to construct the ‘target’ variable for the model, enabling an assessment of its predictive performance. The model was designed to predict whether a given pipe experienced at least one failure during the study period of 20 years. The dataset obtained from the utility included the entire distribution network in Skåne County, Sweden. However, the training and testing datasets were carefully separated based on spatial boundaries. The ML models were trained on the network data, explicitly excluding the case study section, which was specifically set aside for testing. Hence, the model was tested only on the case study section of the distribution network. A notable idea explored by Daulat, et al.^52^, investigated whether ML models trained on data from large or multiple utilities could be used to predict pipe failures in smaller, separate utilities. The approach used in this study adopts a similar idea within a single WDN by spatially separating the training and testing data.

The dataset was preprocessed by removing missing/erroneous records, where approximately 6.5% of records were removed, mainly due to missing core inventory attributes (e.g., installation year, diameter, or length). Inconsistent observations (e.g., failures recorded before installation) and extreme outliers (e.g., diameter = 0) were also filtered. Moreover, since pipe inventory and break records were spatially matched, a small number of break events that could not be matched to any pipe segment were excluded.

Numerical variables were normalized using z-score standardization, with scaling parameters estimated from the training data only to avoid information leakage. A total of three categorical variables (material, roads and trainlines, and soil type) were one-hot encoded^53^. WDNs commonly exhibit imbalanced datasets, as majority of pipes have never experienced a break. This imbalance makes model training difficult because the model struggles to learn patterns associated with the minority class (pipes that have experienced breaks) resulting in poor predictive performance^13,54^. In the present dataset, the positive (break) class represented approximately 7% of samples. To balance the dataset, random oversampling method^55^ was applied only on the training dataset.

##### Model training, hyperparameters tuning and selection

Model training and hyperparameter tuning were performed only on the training dataset using stratified *k*-fold cross-validation to maintain a similar break/non-break class proportion in each fold. Hyperparameters for LR, RF, and XGBoost were optimized using randomized search with area under the precision-recall curve (PR-AUC) as the primary scoring metric, which is more suitable than accuracy for imbalanced classification. Model selection was performed using training-only out-of-fold (OOF) PR-AUC to avoid test-set bias. The selected model was then refit on the full training set and evaluated once on the test case study section. The hyperparameter search ranges are summarized in Table 2, and the complete search ranges are reported in Supplementary Table S1.

**Table 2:** Summary of hyperparameters of ML models.

Model	Hyperparameters	Type	Range
LR	Solver	Categorical	lbfgs, saga
	Penalty	Categorical	lbfgs: l2; saga: l1, l2, elasticnet
	Regularization strength	Float (log-uniform)	[10^− 4^, 10^2^]
	L1 Ratio (if elasticnet)	Float (uniform)	[0.05, 0.95]
RF	Number of estimators	Integer	[30, 300]
	Maximum depth	Integer	[2, 6]
	Maximum features	Categorical	sqrt, log2
	Minimum samples leaf	Integer	[1, 8]
XGBoost	Number of estimators	Integer	[30, 300]
	Learning rate	Float (log-uniform)	[0.01, 0.1]
	Maximum depth	Integer	[2, 6]
	Feature subsampling	Float (uniform)	[0.7, 1.0]

##### Metrics for model evaluation

Model performance was evaluated using both threshold-independent and threshold-dependent metrics. Threshold-independent metrics were used for model benchmarking and selection and included the area under the receiver operating characteristic curve (ROC-AUC) and the PR-AUC. ROC-AUC summarizes class discrimination across all probability thresholds, while PR-AUC summarizes the precision-recall trade-off and is particularly informative under class imbalance. To quantify uncertainty, 95% confidence intervals for ROC-AUC and PR-AUC were estimated on the case study test set using bootstrap resampling.

**Fig. 2:**
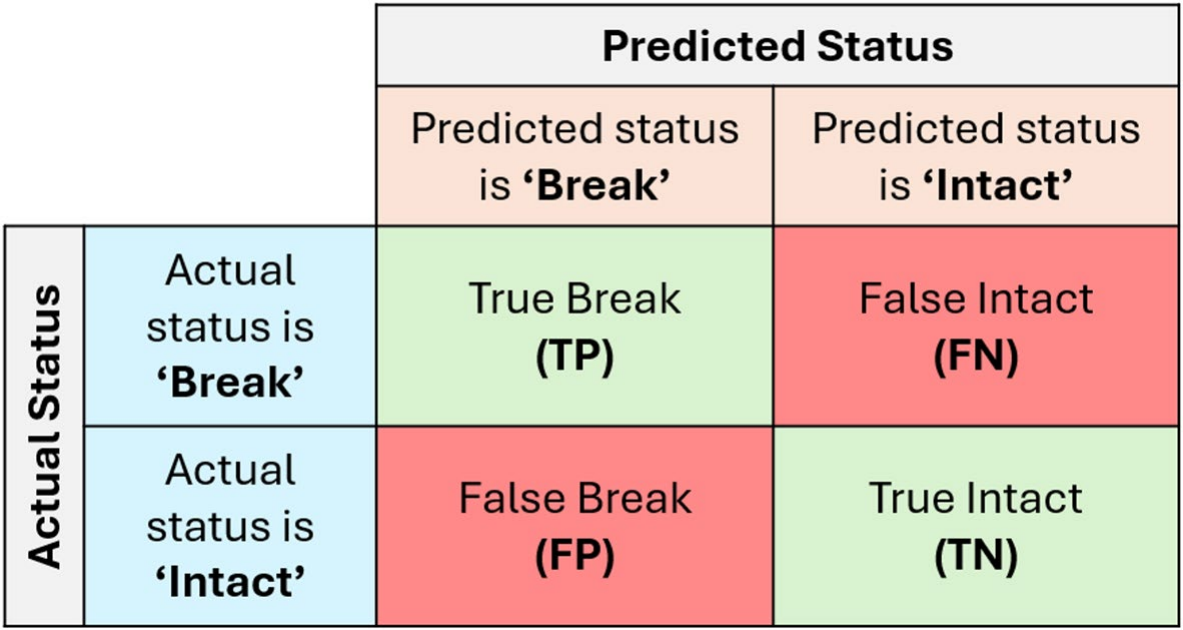
Confusion matrix for pipe status classification.

Threshold-dependent metrics were computed from the confusion matrix (Fig. 2), which can be used to evaluate the model’s performance^56^. A confusion matrix contains the number of true positive (TP), false positive (FP), true negative (TN) and false negative (FN) samples as a result of comparing the true and the predicted labels for all the test samples. The term positive is associated with class 1 ‘break’ and the term negative with class 0 ‘intact.’

Based on the confusion matrix, the performance of the model can be evaluated using metrics such as accuracy, precision, recall, and F1-score. However, for datasets with class imbalance, accuracy may not provide a reliable measure of model performance, as it can be heavily biased toward the majority class. Hence, precision, recall, and F1-score are more reliable metrics for evaluating the model performance. The metrics are defined below:

6 $$\:Accuracy=\frac{TP+TN}{TP+TN+FP+FN}$$

7 $$\:Precision=\frac{TP}{TP+FP}$$

8 $$\:Recall=Sensitivity=\frac{TP}{TP+FN}$$

9 $$\:F1\:score = 2 \cdot \frac{{Precision \cdot Recall}}{{Precision + Recall}}$$

#### Hydraulic consequence

The Water Network Tool for Resilience (WNTR) version 1.3.2^57^ was used to simulate and analyse the hydraulic impacts of pipe breaks within the WDN. Hydraulic consequence analysis was performed using the WNTR Simulator under pressure-driven demand (PDD) conditions over a 24-hour period and the Wagner equation^58^ was used to determine nodal demands based on available pressure.

10 $$\:q{D}_{i}=\left\{\begin{array}{c}{D}_{i},\:\:\:\:\:\:\:\:\:\:\:\:\:\:\:\:\:\:\:\:\:\:\:\:\:\:\:\:\:\:\:\:\:\:\:\:\:\:\:\:\:{p}_{i}\ge\:{P}_{f},\\\:{{D}_{i}\left(\frac{{p}_{i}-{P}_{0}}{{P}_{f}-{P}_{0}}\right)}^{\frac{1}{\epsilon\:}},\:\:\:\:\:\:\:\:\:{P}_{0}<{p}_{i}<{P}_{f},\\\:0,\:\:\:\:\:\:\:\:\:\:\:\:\:\:\:\:\:\:\:\:\:\:\:\:\:\:\:\:\:\:\:\:\:\:\:\:\:\:\:\:\:\:\:{p}_{i}\le\:{P}_{0}\end{array}\right.$$

where *qD*_*i*_ is the actual demand at node *i*, *D*_*i*_ is the nominal demand at node *i*, *p*_*i*_ is the pressure at node *i*, *P*_*0*_ is the minimum pressure below which the demand is zero, *P*_*f*_ is the pressure required to fully satisfy the demand *D*_*i*_, and 1/*ε* is the pressure function exponent usually set to 0.5 modelling flow through orifices or pipes^16^.

Hydraulic consequence was measured by enumerating nodes experiencing pressure below a critical threshold (1 m) after each pipe closure simulation. Nodes already experiencing low baseline pressure were excluded. Results from these simulations were normalized and visualized, highlighting critical pipes whose failures cause widespread low-pressure scenarios.

#### QMRA and health consequence

The QMRA and health consequence analysis were also conducted using WNTR version 1.3.2. The network model was configured with a duration of 48 h, a hydraulic timestep of 1 h, and a quality timestep of 5 min. The demand model was set to PDD. For each pipe in the network, a break was simulated individually by splitting the pipe and introducing a local intrusion point at the new node. No pipe closures or segment-level isolations were applied; thus, only one pipe was affected at a time while adjacent pipes remained in service. The intrusion volume was estimated using the orifice-based formulation in Eq. (11) based on nodal pressure and orifice discharge parameters.

11 $$\:{d}_{leak}=\:{C}_{d}A{p}^{\alpha\:}\sqrt{\frac{2}{\rho\:}}$$

where *d*_*leak*_ is the leak demand, *C*_*d*_ is the discharge coefficient, *A* is the area of the hole, *p* is the gauge pressure inside the pipe, *α* is the pressure exponent, and *ρ* is the density of the fluid. The discharge coefficient was set to 0.6. The pressure exponent was set at the common value of 0.5.

Random values for dilution factor (*D*), frequency factor (*F*), and pathogen concentration in faeces (*C*_*p*_) were generated using specified distributions: uniform (10^− 2^, 10^− 4^) for dilution factor, uniform (0.01, 1) for frequency and triangular (10, 10^2^, 10^3^) for pathogen concentration in faeces. The intrusion mass (*M*) was then calculated as:

12 $$M{\text{ }} = {\text{ }}V \cdot C_{p} \cdot D \cdot F$$

The mass was introduced into the downstream node of the broken pipe, and a hydraulic and water quality simulation was performed. A conservative tracer approach was adopted, meaning that the contaminant was transported without decay or reaction with disinfectant residuals, representing a worst-case contamination scenario. The results were analysed to determine contamination levels at various nodes.

After the quality simulation, the probability of infection (*P*_*inf*_) is calculated based on the contamination levels. The dose (*D*) is calculated as the product of concentration and volume

13 $$\:D = C_{{node}} \cdot V\:\:$$

where *D* is the dose, *C*_*node*_ is the pathogen concentrations at the consumer tap and *V* is the volume of water ingested per person.

The Exact Beta-Poisson model was used to assess the dose-response for each reference pathogen. Using this dose-response model, the probability of infection was calculated with:

14 $$\:{\mathrm{P}}_{{{\mathrm{inf}}}} \: = \:1 - exp^{{ - r\: \cdot \:D}} \:$$

where *P*_*inf*_ is the probability of infection, *r* is a sample from the Beta distribution with *α*,* β* parameters for each pathogen and *D* is the dose ingested.

Pathogen intrusion concentrations were based on E. coli levels as indicators of faecal pollution, using established literature and field data^59,60^. Reference pathogens chosen were *Campylobacter*, norovirus, and *Cryptosporidium*, which represent typical Swedish WDN risks^61^.

The Beta distribution parameters for each reference pathogen were: *Campylobacter* (α = 0.024, β = 0.011)^62^, norovirus (α = 0.04, β = 0.055)^63^ and *Cryptosporidium* (α = 0.115, β = 0.176)^64^. The population was assumed to be homogenous, i.e., all consumers would respond according to the chosen dose-response function. Results were aggregated into mean risks and percentiles, enabling ranking of pipes by infection risk severity.

#### TOPSIS for decision making

##### Development of decision strategies and evaluation criteria

Following the risk assessment, several strategies for reducing health risks through breakage and leakage control were identified based on extensive literature review and structured stakeholder discussions. Ten industry experts, comprising water specialists, utility managers, and water supply consultants, participated in the evaluation. These experts were selected based on their experience and expertise in water distribution system maintenance and rehabilitation in Sweden. The study was carried out in accordance with relevant guidelines and regulations. Ethical approval was not required under the Swedish ethical review framework, as the stakeholder input involved only professional judgments and did not involve any intervention or the collection/processing of sensitive personal data. Informed consent was obtained from all participants.

To facilitate the evaluation process, each expert received an evaluation spreadsheet detailing the various risk-reduction strategies alongside clearly defined evaluation criteria. The identified strategies are shown in Table 3 and the evaluation criteria are shown in Table 4. Stakeholders were requested to provide their judgment by assigning scores on a scale ranging from 1 to 5. On this scale, a score of 1 indicated that the expert considered the strategy the least favourable with respect to the specific criterion, whereas a score of 5 indicated the highest favourability. The expert evaluations were used to prepare a decision matrix, where each row represents a strategy, and each column represents an evaluation criterion.

$$Decision{\mathrm{~}}Matrix = \left( {\begin{array}{*{20}c} {Strategy_{1} } & {E_{1} } & {E_{2} } & {E_{3} } & {E_{4} } & {E_{5} } \\ {Strategy_{1} } & {E_{1} } & {E_{2} } & {E_{3} } & {E_{4} } & {E_{5} } \\ \vdots & \vdots & \vdots & \vdots & \vdots & \vdots \\ {Strategy_{8} } & {E_{1} } & {E_{2} } & {E_{3} } & {E_{4} } & {E_{5} } \\ \end{array} } \right)$$

**Table 3:** Different strategies for reducing risks in WDNs.

Strategies	Strategy description
Pressure management	Regulate water pressure through pressure-reducing valves.
Pipe repair and rehabilitation	Conduct repairs and apply techniques such as pipe lining.
Pipe replacement/renewal	Replace old or deteriorating pipes with new ones (pipe renewal).
Increase inspection and testing	Enhance the frequency and thoroughness of pipe inspections.
Increase metering	Install additional meters to better monitor water flow and usage.
Active leakage control	Implement strategies for continuous measurement and management of leaks, including the use of specialized tools.
Cross connection control	Implement measures to identify and manage unauthorized or improper cross-connections.
Public awareness and engagement	Educate and engage the community on the importance of leak reporting.

**Table 4:** Evaluation criteria for different strategies.

Evaluation criteria (E)	Criteria description
Cost	The financial expenditure required to implement and maintain the strategy.
Executability	The ease and feasibility of implementing the strategy within the existing infrastructure.
Risk reduction	The extent to which the strategy lowers the overall risk.
Social concern	The strategy’s impact on the community and stakeholders (social, environmental and public health).
Reliability	The consistency and dependability of the strategy over time.

##### TOPSIS analysis

The TOPSIS method was applied to rank the identified strategies. TOPSIS identifies the best alternative as the closest to an Ideal Solution (IS) and furthest from a Negative Ideal Solution (NIS). The analysis involved the following steps:

##### Normalization of the decision matrix

Criteria scores were normalized using vector normalization to ensure comparability:

15 $$\:{r}_{ij}=\frac{{x}_{ij}}{\sqrt{{\sum\:}_{i=1}^{n}{x}_{ij}^{2}}}$$

where *x*_*ij*_ is the score of strategy *i* for criterion *j*, *n* is the number of strategies and *r*_*ij*_ is the normalized value.

##### Calculation of ideal and negative ideal solutions

The IS and NIS are defined as follows:

16 $$\:{v}^{+}=(\mathrm{max}\left({v}_{1j}\right),\mathrm{max}\left({v}_{2j}\right),\dots\:\:,\mathrm{max}\left({v}_{nj}\right))$$

17 $$\:{v}^{-}=(\mathrm{min}\left({v}_{1j}\right),\mathrm{min}\left({v}_{2j}\right),\dots\:\:,\mathrm{min}\left({v}_{nj}\right))$$

In Eq. (16) and Eq. (17), *v*_*ij*_ is the value for strategy *i* and criterion *j*, *v*^*+*^ is the ideal solution, taking the maximum value for each criterion across all strategies and *v*^*−*^ is the negative ideal solution, taking the minimum value for each criterion across all strategies.

##### Calculation of Euclidean distances

For each strategy, the distance from both the ideal best and ideal worst solutions was calculated using Euclidean distance.

18 $$\:{S}_{i}^{+}=\sqrt{\sum\:_{j=1}^{n}{\left({v}_{ij}-{v}_{j}^{+}\:\right)}^{2}}$$

19 $$\:{S}_{i}^{-}=\sqrt{\sum\:_{j=1}^{n}{\left({v}_{ij}-{v}_{j}^{-}\:\right)}^{2}}$$

where *S*_*i*_^*+*^ and *S*_*i*_^*−*^ are the distances from the IS and NIS, respectively.

##### TOPSIS score calculation

The TOPSIS score for each strategy is calculated using the Relative Closeness *(RC*_*i*_*)*, which measures how close a strategy is to the IS while accounting for its distance from the NIS. Strategies with higher TOPSIS scores represent more favourable alternatives.

20 $$\:{RC}_{i}=\frac{{S}_{i}^{-}}{{S}_{i}^{+}+{S}_{i}^{-}}$$

To assess uncertainty in the MCDA inputs, a bootstrap analysis was performed by resampling the experts with replacement and recomputing the normalized criterion weights and corresponding TOPSIS scores for each replicate. The resulting bootstrap distributions were used to report 95% uncertainty intervals for the criterion weights and TOPSIS scores.

## Results and discussions

### Probability assessment of the case study

The first step of the analysis involved identifying vulnerable pipes by utilizing the ML models. As described in Sect. "Break Probabilities", all ML models were trained while explicitly excluding the case study section, which was reserved for evaluation purposes. Based on the training-only OOF PR-AUC criterion, RF was selected as the final predictor. Table 5 summarizes the discrimination performance of all ML models on the case study section using ROC-AUC and PR-AUC, with 95% bootstrap confidence intervals.

**Table 5:** Performance of ML models on the case study section.

Model	ROC-AUC (95% CI)	PR-AUC (95% CI)
RF	0.897 [0.82, 0.95]	0.645 [0.48, 0.79]
XGB	0.883 [0.79, 0.95]	0.635 [0.47, 0.78]
LR	0.858 [0.75, 0.94]	0.595 [0.43, 0.73]

Due to security reasons, detailed visualizations and performance results are presented only for the case study section, summarized in Table 6, which reports the corresponding confusion matrix and threshold-dependent performance metrics for the RF model. The decision threshold for RF (0.36) was chosen using training-only OOF predictions to meet a minimum precision of 0.50 and was then applied unchanged to the case study section.

**Table 6:** Confusion matrix and evaluation metrics on the case study section.

		Predicted status	
		Predicted ‘break’	Predicted ‘Intact’
Actual Status	Actual ‘Break’	21	14
	Actual ‘Intact’	18	402

Evaluation metrics of the RF model (with threshold: 0.36)			
Accuracy	Precision	Recall	F1-score
0.93	0.54	0.6	0.57

Despite the limited test set size, the RF model showed reasonable performance in classifying failure-prone pipes. The model was able to identify 60% of the actual pipes that suffered a break during the study period over the past 20 years. Similarly, the model was able to classify 96% of the pipes not experiencing a break. The model precision was 54%, accuracy 93%, and the resulting F1-score was 57%.

To provide additional insight, permutation importance was evaluated for the RF model as the decrease in average precision resulting from randomly permuting each input feature. The results showed that age was the most influential variable, followed by material and length. This is consistent with findings reported in previous studies, which have similarly identified physical pipe characteristics as the most influential features in failure prediction^65–67^. Soil type, Impact score, diameter, and traffic intersection exhibited lower and broadly similar importance. These findings indicate that the model was driven mainly by physical pipe-related characteristics, although the observed importance patterns should be interpreted in the context of the relatively small case study section.

The model’s output represents the model’s confidence in classifying a pipe as belonging to the category of historically failed pipes. For pipes with no prior failures, a high predicted probability indicates that they share similar characteristics with failed pipes, suggesting increased vulnerability and a greater risk of future breaks. While these probability values do not represent absolute failure predictions, they provide a quantitative basis for assessing relative vulnerability across the network, thereby supporting the prioritization of maintenance efforts. Improving the model’s predictive accuracy was not the primary focus of this study, as the model was intended to serve just as an intermediate step within the broader risk assessment framework.

The predicted break probabilities were calibrated so that the numerical probability values better reflect observed event frequencies. Specifically, the RF output scores were post-processed using a sigmoid calibration model fitted on the training data only (via cross-validation) and then applied unchanged to the case study section. The calibrated probabilities were subsequently combined with consequence values to calculate overall pipe-level risk. Furthermore, the results were visualized using a GIS-based map to support spatial interpretation.

**Fig. 3:**
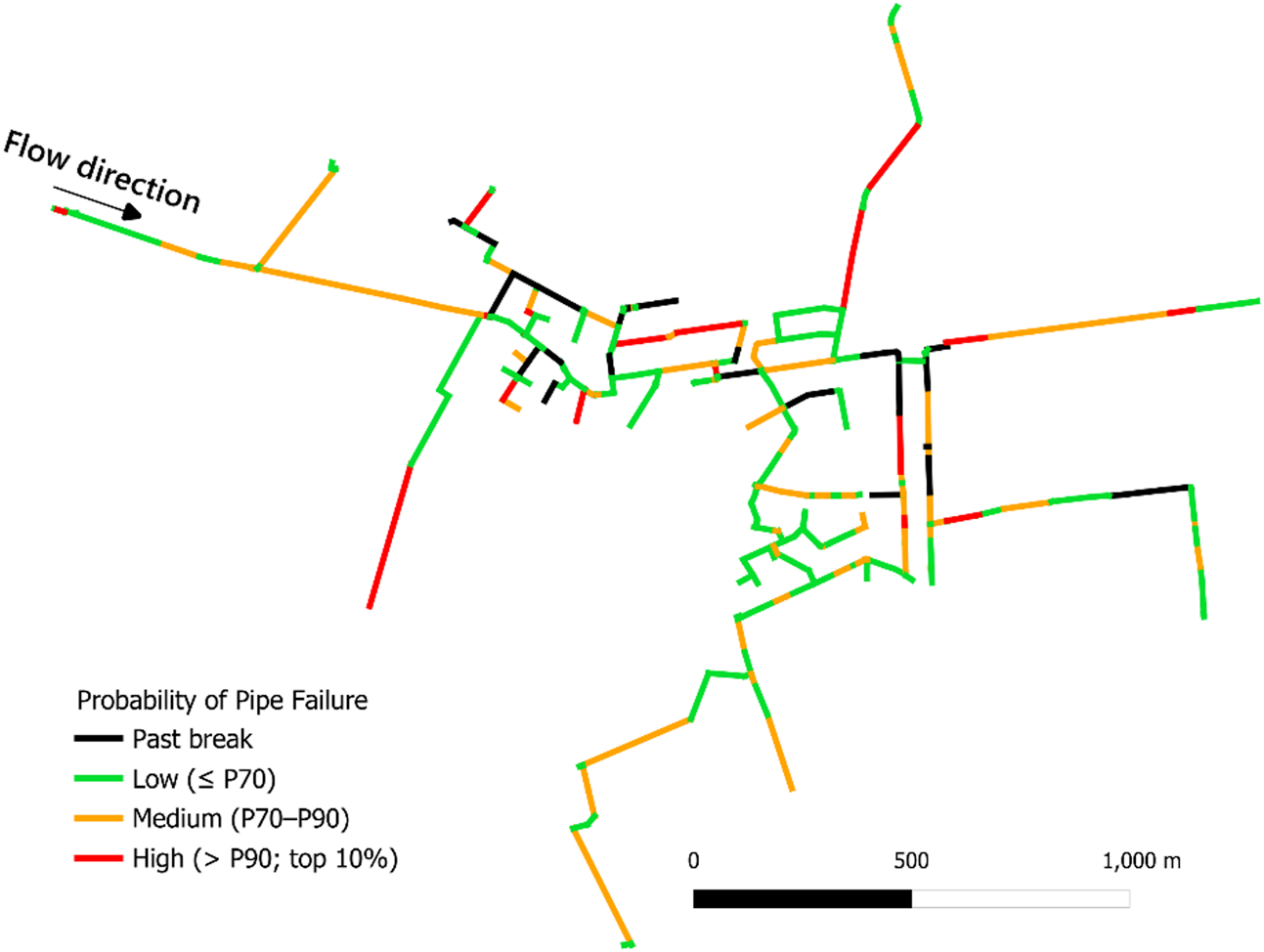
Map of the WDN showing RF-predicted break vulnerability. Pipes are grouped into relative vulnerability tiers based on the distribution of predicted probabilities: lower vulnerability (≤ P70; bottom 70%), moderate vulnerability (P70–P90; next 20%), and higher vulnerability (> P90; top 10%). Past breaks are shown in black.

The predicted probabilities were skewed toward smaller values, making direct mapping using fixed probability cut-offs less informative. Therefore, a relative vulnerability (quantile-based) classification was applied so spatial patterns are interpreted in terms of ranked vulnerability rather than absolute probability levels. Pipes were grouped into three tiers based on the predicted-probability distribution: Lower vulnerability (≤ 70th percentile; bottom 70%), Moderate vulnerability (70th–90th percentile; next 20%), and Higher vulnerability (> 90th percentile; top 10%). Figure 3 illustrates this spatial mapping. For security reasons, details in the map have been removed and the network configuration have been intentionally distorted.

### Consequence assessment of the case study

#### Hydraulic consequence

**Fig. 4:**
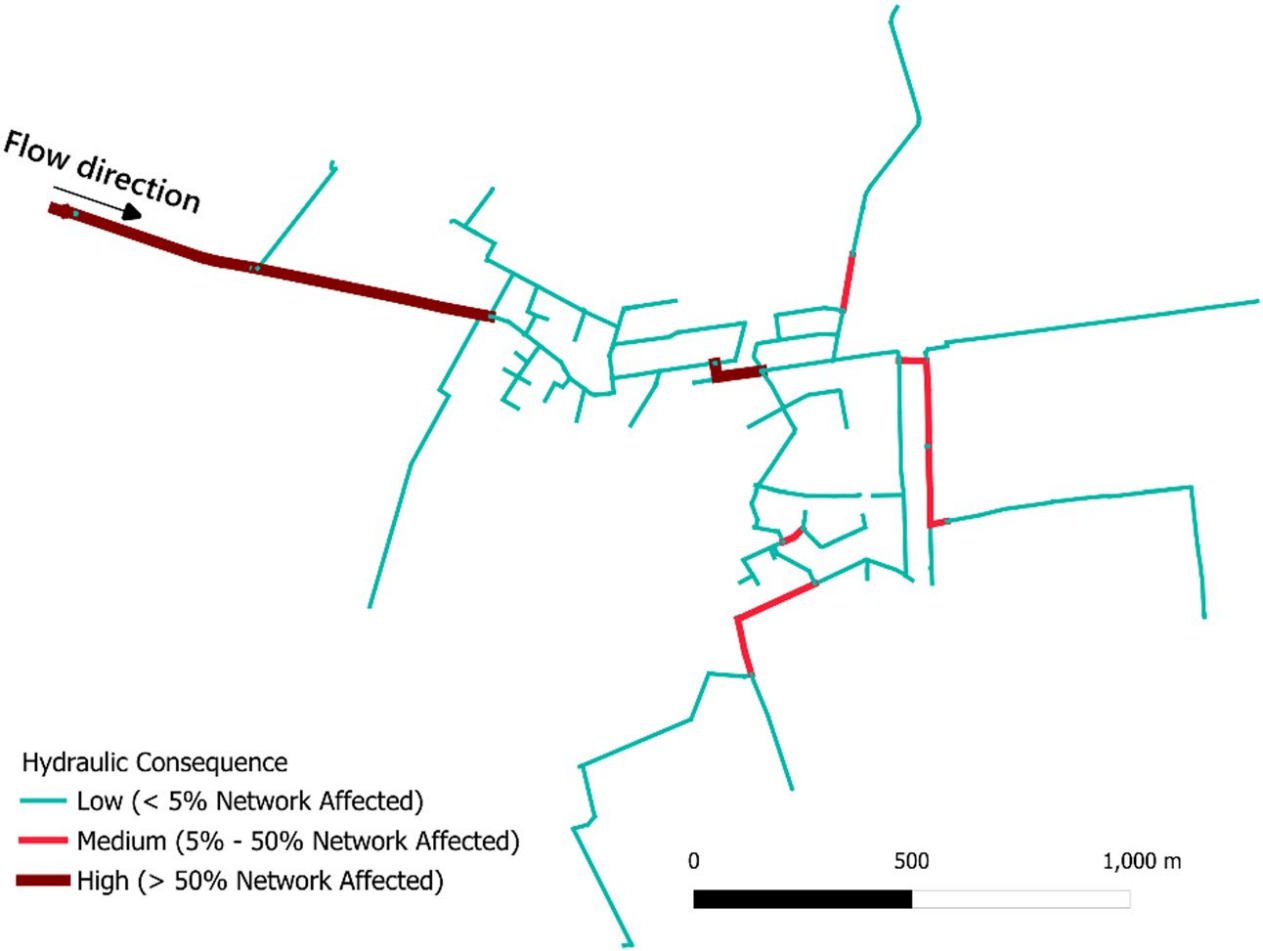
Map of the WDN showing pipe segments categorized by the hydraulic disruptions they would cause in the event of a pipe break.

The hydraulic consequence, i.e., impact of a pipe break on the overall system flow and pressure, is shown in Fig. 4. Pipes with high consequence scores are the ones that have a large impact on the hydraulic performance of the network. These pipes are often found in critical locations such as trunk lines feeding large subnetworks, or single-feed pipelines supplying elevated zones. In this study, pipes were categorized into three consequence levels: low (affecting < 5% of the network), medium (affecting 5–50% of the network), and high (affecting > 50% of the network). These thresholds were selected to provide a clear and interpretable visualization of the network’s hydraulic vulnerability. However, these thresholds are adjustable as per utility’s own operational standards, risk tolerance, or service-level objectives.

In the case study section, majority of pipe segments exhibited low hydraulic consequence, suggesting that their failure would result in only localized service disruptions. However, a small number of critical mains were associated with high consequence scores, indicating that their failure would lead to significant pressure drops or widespread service interruptions across large areas of the WDN. Notably, one of these high-consequence pipes corresponds to the segment previously evaluated by Odhiambo, et al.^11^ for intrusion risks. Other important pipes were mainly located in the central part of the network, branching out from the grid loop. The geography and layout of the network, together with its connectivity to critical demand nodes and the availability of redundant flow paths were found to strongly influence the hydraulic consequence of failures.

#### Health consequence

**Fig. 5:**
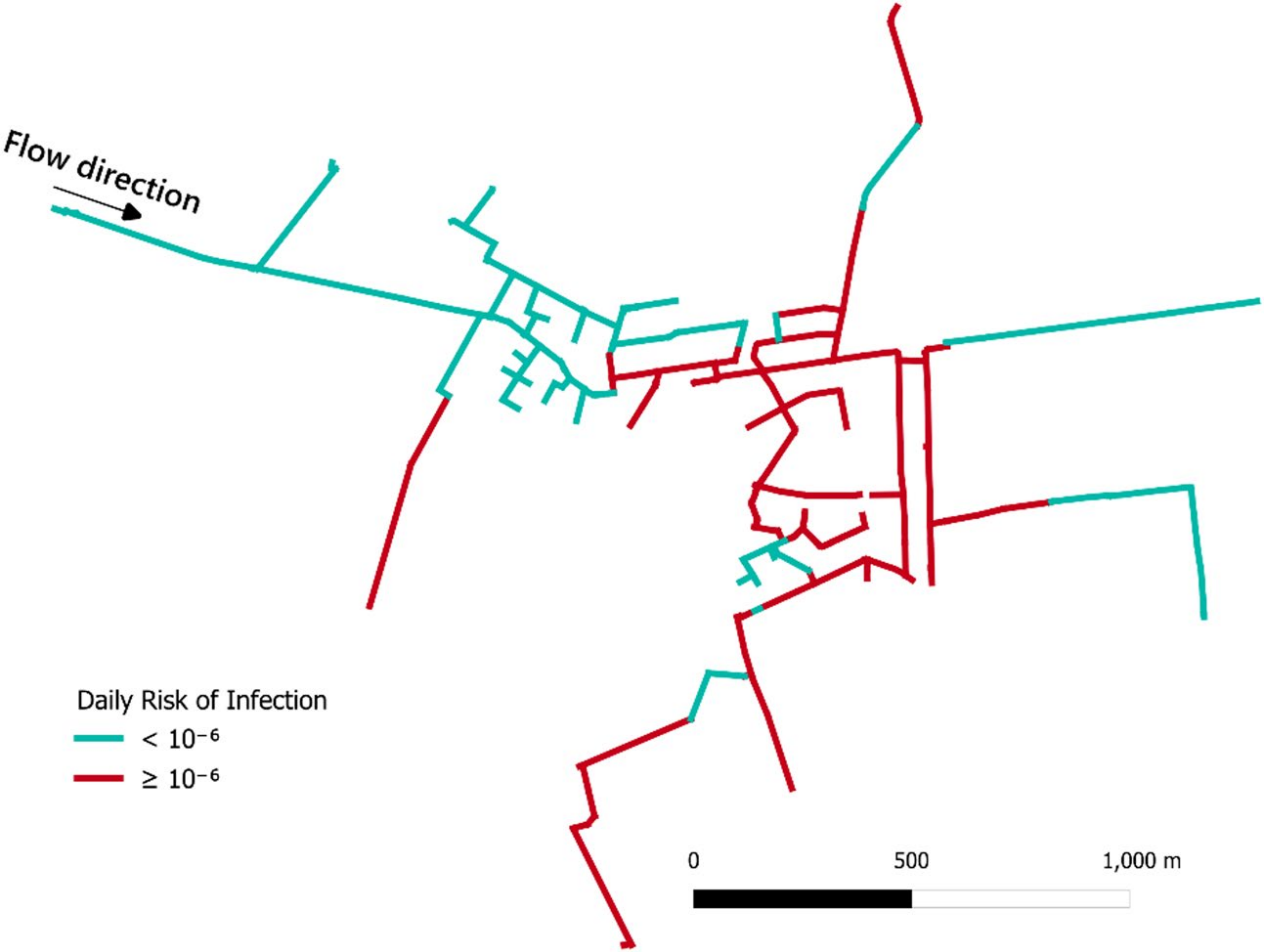
Map of the WDN showing pipe segments with a daily risk of infection above 10^− 6^ in red.

The health consequence, obtained after calculating the daily risk of infection, is presented in Fig. 5. Pipe segments were classified into two categories based on the estimated risk: those with a daily infection risk exceeding 10^− 6^ and those below the 10^− 6^ threshold. The acceptable risk target for daily probability of infection was set at 10^− 6^, to account for short-term periods of heightened risk^68^.

The WDN segments marked in red represent areas that, on average, exceed the acceptable daily risk target throughout the simulation. The spatial pattern of Fig. 5 reveals that certain pipes, often the same ones with high hydraulic impact also rank high in contamination risk. Notably, the highest risks are concentrated in the central section of the WDN and certain branches. This is likely due to a combination of hydraulic conditions, infrastructure vulnerabilities, network topology, and intrusion-related source assumptions (e.g., pathogen concentration and dilution factors). Although other WDNs may behave differently, this approach provides a valuable means of identifying and visualizing pipes with respect to potential health consequences.

### Risk assessment of the case study

Risk assessment of the pipes in the distribution network was conducted by combining the calibrated probability (P) values, obtained from the RF model, with the corresponding normalized consequence (C) values, derived from both hydraulic and health consequence calculations, using Eq. (2). The calibrated probability values ranged from 0 to 1, while the hydraulic and health consequence values were normalized to a common range of 0 to 1. Thus, the risk scores represent both the probability of failure and the severity of its potential consequences. These values were then visualized spatially using a GIS-based mapping platform, enabling clear, color-coded differentiation of risk levels across the network.

**Fig. 6:**
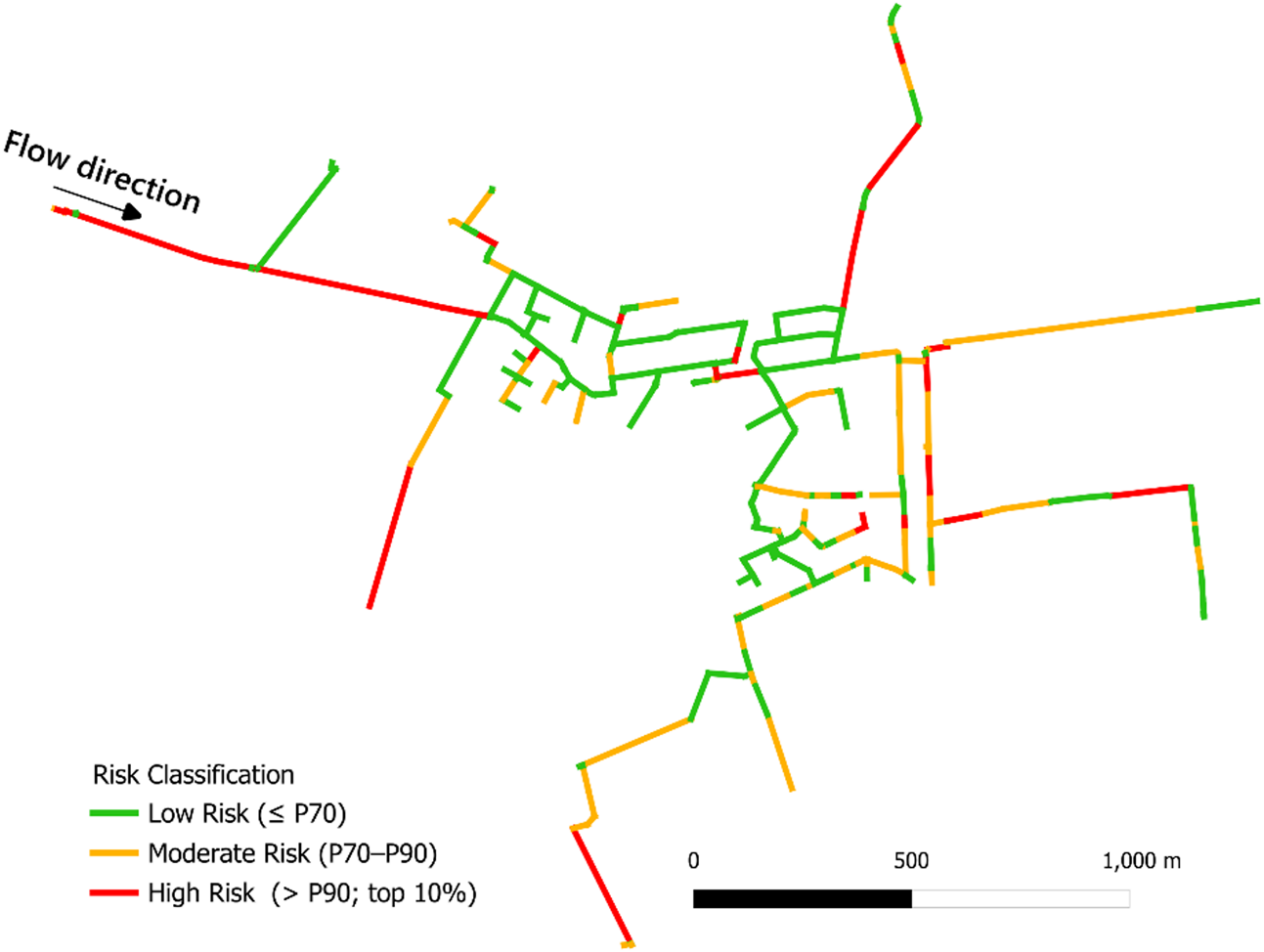
Map of the WDN showing pipe segments with total risk.

Spatial risk maps facilitate intuitive interpretation, helping utilities identify high-risk zones for targeted maintenance or replacements. Previous studies^69,70^ have similarly used spatial mapping in WDNs. Figure 6 presents the spatial distribution of pipe risk classifications within the case study section, highlighting low-risk (bottom 70%), moderate-risk (next 20%), and high-risk (top 10%) segments. The classification was rank-based, based on the distribution of pipe-level risk scores. Accordingly, the three groups corresponded to 6.3 km, 4.1 km, and 3.1 km of pipe, respectively. The resulting map provides a spatial representation of relative risk levels among pipes, enabling comparison of potential vulnerabilities across the network. This can assist water utilities in prioritizing pipe inspection, monitoring, maintenance, and rehabilitation efforts, with pipes in the highest risk tier representing priority candidates for further assessment and targeted intervention, while low-risk pipes can be deprioritized or monitored.

### TOPSIS results

The normalized weights of evaluation criteria, derived from expert input, are presented in Table 7. These weights reflect the relative importance assigned to each criterion within the decision-making process. The results indicate that risk reduction was prioritized most strongly by the experts, highlighting a strong emphasis on minimizing potential health risks and ensuring public safety. Executability and reliability were also highly valued, highlighting the significance of strategies that are feasible and dependable. Cost and social concern received comparatively lower weights but remained non-negligible contributors to the overall decision process. Criteria weights were derived from the expert ratings by first averaging the criterion scores across experts and then normalizing them so that the weights sum to 1. To reflect uncertainty in expert-based judgments, 95% uncertainty intervals were estimated by bootstrap resampling of experts with replacement and recomputing the normalized criterion weights.

**Table 7:** Normalized weights based on expert scores.

Criterion	Ranking	Weight (95% CI)
Risk reduction	1	0.266 [0.24, 0.29]
Executability	2	0.201 [0.18, 0.22]
Reliability	3	0.200 [0.17, 0.22]
Cost	4	0.174 [0.14, 0.20]
Social concern	5	0.157 [0.13, 0.18]

**Fig. 7:**
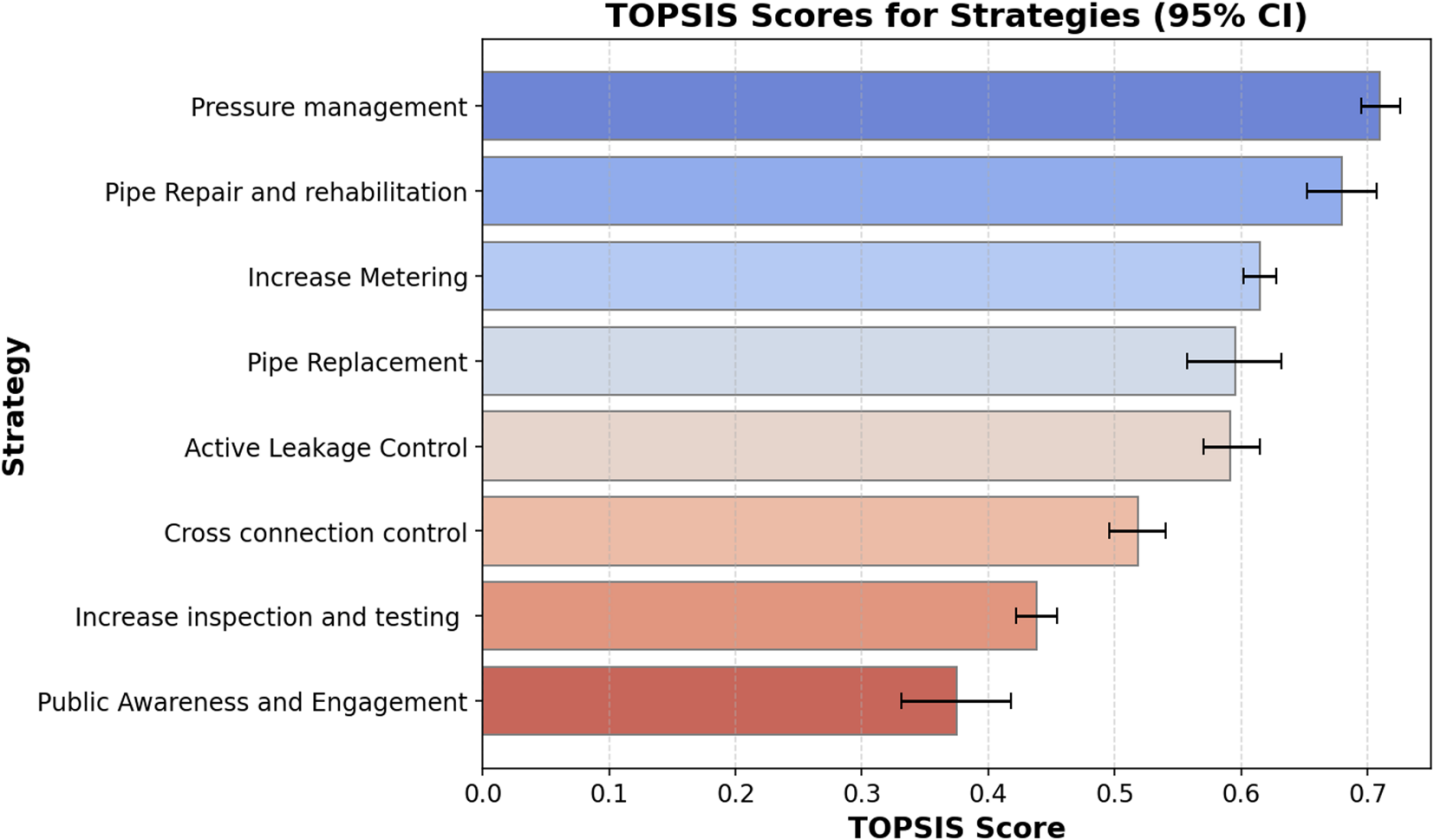
Final TOPSIS scores for each alternative (mean expert weights with bootstrap 95% uncertainty intervals).

Figure 7 presents the results obtained from the TOPSIS method for strategy prioritization. The ranking highlights ‘Pressure Management’ as the most effective strategy with the highest TOPSIS score (0.71), followed by ‘Pipe Repair and Rehabilitation’ (0.67) and ‘Increase Metering’ (0.61). Strategies such as ‘Pipe Replacement,’ ‘Active Leakage Control’ and ‘Cross-Connection Control’ were ranked in the middle, while ‘Increase inspection and testing’ and ‘Public Awareness and Engagement’ ranked lowest, indicating a lower perceived impact towards health risk reduction.

Expert inputs produced a structured prioritization, underscoring proactive measures over reactive approaches. Pressure management achieved the highest overall score due to its strong performance across multiple criteria. It involves regulating network pressure using methods such as pressure-reducing valves or optimizing pump schedules. This strategy is cost-effective, easily executable, and significantly reduces network-wide risks. This outcome aligns with prior research showing that pressure management can substantially reduce burst frequencies and leakage volumes, while requiring less investment than full pipe replacements^71,72^. Pipe repair and rehabilitation also ranked high, especially regarding risk reduction and reliability, but scored lower in cost and executability, slightly impacting its overall ranking.

Strategies such as active leakage control and increased metering were rated moderately effective due to ease of implementation and lower costs, but they offer less direct risk reduction compared to physical interventions. These results reinforce the value of data-driven, preventive management approaches that proactively mitigate failures. Nevertheless, reactive strategies like pipe replacement remain essential as complementary long-term measures. The findings align with contemporary risk management principles in water supply systems and underscore the importance of balancing technical feasibility, cost-effectiveness, and social considerations to ensure that proposed solutions are both practical and acceptable to stakeholders.

As a robustness check, a weighted sum model (WSM) was also applied using the same criterion weights. In WSM, each strategy receives a score by first normalizing its criterion ratings to a common scale (0–1) and then computing the weighted sum across criteria, so strategies performing well on highly weighted criteria obtain higher overall scores^73^. The WSM-based ranking was compared with the TOPSIS ranking using Spearman’s rank correlation. Spearman’s rank correlation coefficient (*ρ*) measures the degree of agreement between two rankings by correlating the rank positions of the strategies; values close to 1 indicate very similar rankings, values near 0 indicate little agreement, and negative values indicate opposite ordering. The TOPSIS and WSM rankings showed strong agreement (*ρ* = 0.85), indicating that the main prioritization trends were not sensitive to the choice of MCDA aggregation method.

The final TOPSIS rankings are sensitive to the assigned criteria weights. For instance, utilities prioritizing safety might rank pipe replacement higher, whereas those operating under budget constraints may favour cost-effective solutions. The flexibility of TOPSIS accommodates varying organizational goals, risk tolerance, and resource availability, and demonstrates its strength as a robust decision-support method adaptable to diverse utility contexts.

## Conclusions and further work

This study demonstrates an integrated approach to health risk assessment and decision-making within the WSP framework through a case study of a WDN in Sweden. Risk assessment was conducted by integrating ML, QMRA and hydraulic modelling. By applying three ML models (LR, RF, and XGBoost) to estimate the likelihood of pipe breaks and combining it with QMRA and hydraulic modelling, this study effectively identified vulnerable pipes in the WDN. The results were integrated into a GIS framework for the development of a risk map which provided a spatial representation of vulnerability levels within the network.

Expert interviews and stakeholder engagement were utilized to identify key leakage control strategies for health risk reduction, which were subsequently evaluated using MCDA. Results from the TOPSIS method aligned well with WSP goals, prioritizing pressure management and pipe rehabilitation. These findings highlight the significance of investing in infrastructure maintenance and operational efficiency to mitigate health risks and improve network reliability. Employing MCDA also facilitated stronger stakeholder collaboration for risk management, establishing a robust foundation for informed decision-making regarding health risks from pipe failures and ensuring sustainable water supply systems.

The integrated risk results can support different intervention pathways depending on whether risk is driven mainly by probability of failure or consequence of failure. Pipes with high probability but low consequence may be addressed through preventive actions such as inspection, leak detection, pressure monitoring, valve exercising, or targeted maintenance. In contrast, pipes with lower probability but higher consequence may require measures aimed at reducing impacts, such as improved redundancy, isolation capacity, protection of critical customers, or contingency planning. Pipes ranking high in both dimensions may warrant more intensive interventions, including rehabilitation or replacement.

Several areas were identified for further research. Since the case study network is relatively small, the reported performance metrics and prioritization results should not be assumed to generalize beyond the studied context. At the same time, this setting reflects a common reality for many small and medium utilities with relatively few failures and limited data access, and the case study demonstrates that the proposed WSP workflow can still be implemented in practice by linking failure likelihood, consequence modelling, and decision support under realistic data limitations. Expanding the dataset (e.g., additional years, larger service areas, or multiple utilities), together with more systematic model optimization, would strengthen predictive accuracy, enable stronger external validation, and support more robust inferences.

Further improvements are also possible on the predictive modelling side. Incorporating time-dependent variables would enable more accurate forecasting of pipe failures, which is important for proactive planning. While the evaluated ML models performed reasonably, future studies could extend the comparison to additional model families (e.g., neural networks) and adopt robustness-oriented workflows such as nested cross-validation, together with more systematic hyperparameter optimization (e.g., Bayesian optimization). Model ensembling and explicit cost-sensitive learning could also be explored to better reflect utility-specific inspection and rehabilitation constraints and to align model outputs with operational decision-making. In practice, such outputs could support a simple decision workflow in which higher-risk pipes are first identified, followed by selection of the most appropriate intervention strategy using the TOPSIS rankings under local utility constraints and priorities. Additionally, the ‘Impact Score’ feature, despite capturing valuable network interdependencies, requires extensive hydraulic simulations. Future research could explore optimized simulation techniques to enhance computational scalability.

Since pipe-break datasets are typically highly imbalanced, random oversampling was applied in this study to increase the representation of the minority class due to its simplicity. However, oversampling duplicates minority observations and may increase overfitting risk. Future studies could compare alternative imbalance strategies (e.g., class-weight adjustment or synthetic oversampling such as SMOTE) and evaluate their effect on both discrimination and probability quality, particularly when larger datasets are available.

On the consequence and decision-support side, the analysis can be extended to include additional consequence dimensions such as repair costs, service downtime, customer impacts, or proximity to critical infrastructure, providing a more comprehensive basis for pipe-level risk assessment. The MCDA component can also be strengthened by combining expert judgement with available quantitative indicators where possible, periodically updating stakeholder inputs to reflect changing priorities, and validating prioritization outcomes against implemented interventions and observed performance improvements.

The proposed workflow spans multiple sources of uncertainty, including uncertainty in failure likelihood estimates, hydraulic parameters, QMRA inputs, and expert judgement used in MCDA, which can introduce some subjectivity in the criteria weighting. In this study, uncertainty was addressed at two practical decision points: uncertainty bounds were reported for ML discrimination performance, and bootstrap-based uncertainty intervals were computed for expert-derived criteria weights and resulting TOPSIS scores. Building on this, future work can move from component-level uncertainty reporting toward end-to-end uncertainty propagation, quantifying how uncertainty in probability and consequence jointly affects final risk estimates and strategy rankings. Similarly, alternative risk formulations can be explored, to better represent nonlinear effects, cascading failures, and multiple interacting scenarios (e.g., scenario-based analyses, fault-/event-tree logic, or Monte Carlo propagation). Finally, the GIS mapping component was constrained by security requirements, which required removal or distortion of certain cartographic elements. This limits quantitative spatial interpretation, including detailed assessment of network density and the spatial concentration of higher-risk areas. However, the risk map remains useful for decision support by showing relative vulnerability patterns and highlighting priority areas for inspection and intervention.

In conclusion, this study provides a practical demonstration of integrating predictive modelling, consequence evaluation, and structured decision-making within the WSP framework. The application of this approach in a real-world case study illustrates how utilities with limited data can identify vulnerable assets, assess potential health consequences, and prioritize risk mitigation strategies. This approach is adaptable to different network sizes, data availability, and operational contexts, making it useful for a wide range of water utilities. By combining technical analysis with stakeholder input, it strengthens risk-based planning and supports more informed decision-making in water supply systems.

## Electronic Supplementary Material

Below is the link to the electronic supplementary material.

Supplementary Material 1.

## Data Availability

The data that support the findings of this study are available from the collaborating water utility in Skåne, Sweden, but restrictions apply to the availability of these data, which were used under licence for the current study and are therefore not publicly available. Data are however available from the corresponding author upon reasonable request and with permission of the water utility.
