# Supplementary material for: Integrating machine learning and multi-criteria decision analysis for health risk management in water distribution networks
Source: Sci Rep. 2026 May 15;16:15718. doi: 10.1038/s41598-026-52465-z (PMC13190735; doi:10.1038/s41598-026-52465-z)
Supplement: Supplementary file 1 — Supplementary Material 1. [file 41598_2026_52465_MOESM1_ESM.docx]

**Integrating Machine Learning and Multi-Criteria Decision Analysis for Health Risk Management in Water Distribution Networks**

**Uchit Sangroula^1, *^, Victor Viñas^1,2^, Michael Odhiambo^3^, Thomas J.R. Pettersson^1^**

^1^ Department of Architecture and Civil Engineering, Water Environment Technology, Chalmers University of Technology, SE-412 96 Gothenburg, Sweden.

^2^ AFRY AB, Grafiska vägen 2A, SE-412 83, Gothenburg, Sweden.

^3^ Department of Urban Water Engineering, Norconsult AB, SE-402 76 Gothenburg, Sweden.

^*^ uchit@chalmers.se.

*Supplementary Table S1: Full hyperparameter search space used for randomized tuning (100 sampled settings per model; 5-fold stratified CV)*

| **Model** | **Hyperparameter** | **Type** | **Range / Options** |
| --- | --- | --- | --- |
| LR | Solver | Categorical | {lbfgs, saga} |
|  | Penalty | Categorical | {lbfgs: l2; saga: l1, l2, elasticnet} |
|  | Regularization strength | Float (log-uniform) | [10^-4^, 10^2^] |
|  | Tolerance | Float (log-uniform) | [10^-6^, 10^-3^] |
|  | Fit intercept | Boolean | {True} |
|  | Elastic-net mixing (l1 ratio) (if elasticnet) | Float (uniform) | [0.05, 0.95] |
| RF | Number of trees | Integer | [30, 300] |
|  | Maximum depth | Integer (set) | [2, 6] |
|  | Minimum samples per leaf | Integer | [1, 8] |
|  | Minimum samples to split | Integer | [2, 16] |
|  | Max features | Categorical | {sqrt, log2} |
|  | Bootstrap sampling | Boolean | {True, False} |
| XGBoost | Number of estimators | Integer | [30, 300] |
|  | Learning rate | Float (log-uniform) | [0.01, 0.1] |
|  | Maximum depth | Integer | [2, 6] |
|  | Subsample | Float (uniform) | [0.7, 1.0] |
|  | Column sampling | Float (uniform) | [0.7, 1.0] |
|  | Minimum child weight | Integer | [1, 12] |
|  | L2 regularization (lambda) | Float (log-uniform) | [10^-2^, 10^2^] |
|  | L1 regularization (alpha) | Float (log-uniform) | [10^-4^, 10^1^] |
|  | Tree split penalty (gamma) | Float (log-uniform) | [10^-5^, 10^0^] |

*Supplementary Table S2. Aggregated evaluation scores (1–5 scale) for each strategy across five criteria used in TOPSIS analysis.*

| **AGG** | **Details** | **C1** | **C2** | **C3** | **C4** | **C5** |
| --- | --- | --- | --- | --- | --- | --- |
| **A1** | Pressure management | 3.6 | 3.5 | 3.5 | 2.8 | 3.3 |
| **A2** | Pipe Repair and rehabilitation | 2.3 | 3.5 | 3.7 | 3.6 | 3.6 |
| **A3** | Pipe Replacement | 1.5 | 2.6 | 4.1 | 3.5 | 4.1 |
| **A4** | Increase inspection and testing | 2.8 | 2.8 | 2.8 | 3.1 | 3.0 |
| **A5** | Increase Metering | 2.9 | 3.9 | 3.0 | 3.3 | 3.4 |
| **A6** | Active Leakage Control | 3.4 | 3.6 | 2.9 | 3.1 | 3.2 |
| **A7** | Cross connection control | 3.1 | 2.3 | 3.2 | 3.5 | 3.1 |
| **A8** | Public Awareness and Engagement | 3.6 | 3.0 | 2.0 | 3.3 | 2.1 |

*C1–C5 represent Cost, Executability, Risk Reduction, Social Concern, and Reliability, respectively*


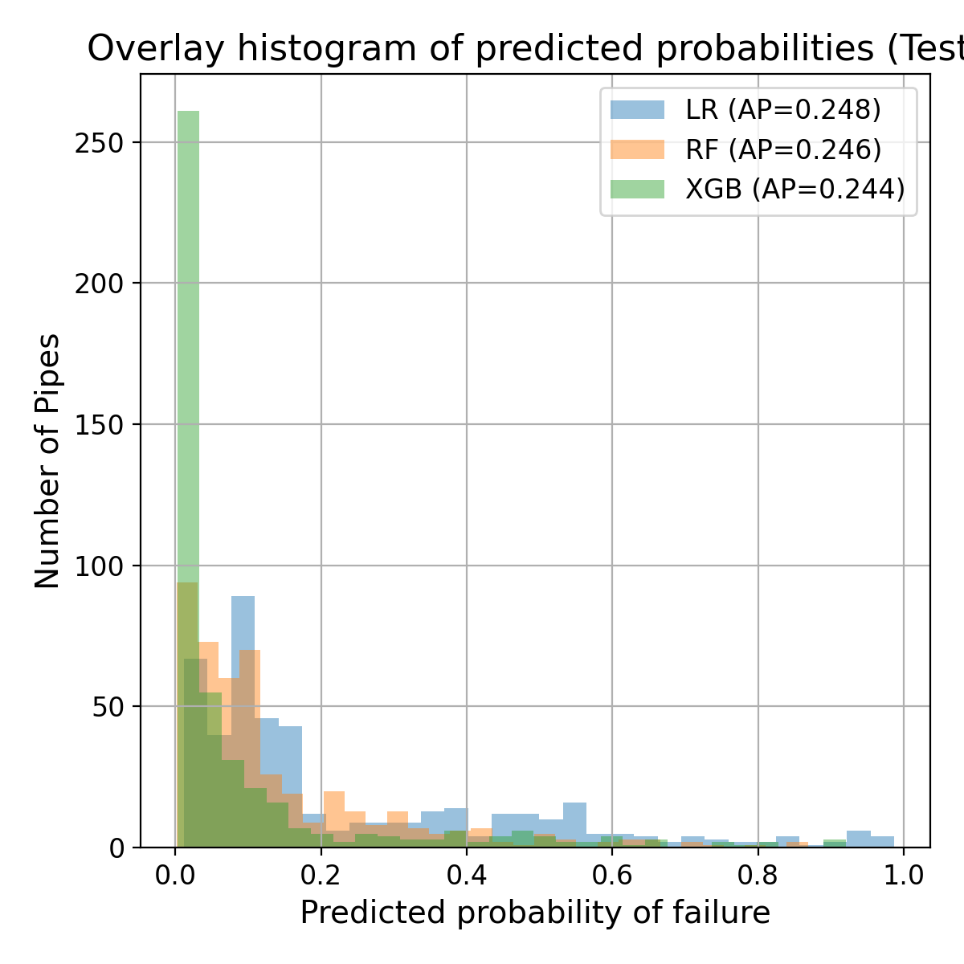


Supplementary Figure 1. Histogram of predicted probabilities from the models for case study section
